# Non-destructive characterisation and classification of ceramic artefacts using pEDXRF and statistical pattern recognition

**DOI:** 10.1186/1752-153X-6-102

**Published:** 2012-09-14

**Authors:** Maja D Gajić-Kvaščev, Milica D Marić-Stojanović, Radmila M Jančić-Heinemann, Goran S Kvaščev, Velibor Dj Andrić

**Affiliations:** 1 Vinča Institute of Nuclear Sciences, University of Belgrade, Mike Petrovića Alasa 12-14, Belgrade, Serbia; 2 National Museum Belgrade, Trg Republike 1a, Belgrade, Serbia; 3 Faculty of Technology and Metallurgy, University of Belgrade, Karnegijeva 4, Belgrade, Serbia; 4 Faculty of Electrical Engineering, University of Belgrade, Bul. kralja Aleksandra 73, Belgrade, Serbia

**Keywords:** pEDXRF spectrometry, Pattern recognition, Dimension reduction, Feature extraction, Classification, Cultural Heritage, Neolithic ceramics

## Abstract

**Background:**

Portable energy dispersive X-ray fluorescence (pEDXRF) spectrometry analysis was applied for the characterisation of archaeological ceramic findings from three Neolithic sites in Serbia. Two dimension reduction techniques, principal component analysis (PCA) and scattering matrices-based dimension reduction were used to examine the possible classification of those findings, and to extract the most discriminant features.

**Results:**

A decision-making procedure is proposed, whose goal is to classify unknown ceramic findings based on their elemental compositions derived by pEDXRF spectrometry. As a major part of decision-making procedure, the possibilities of two dimension reduction methods were tested. Scattering matrices-based dimension reduction was found to be the more efficient method for the purpose. Linear classifiers designed based on the desired output allowed for 7 of 8 unknown samples from the test set to be correctly classified.

**Conclusions:**

Based on the results, the conclusion is that despite the constraints typical of the applied analytical technique, the elemental composition can be considered as viable information in provenience studies. With a fully-developed procedure, ceramic artefacts can be classified based on their elemental composition and well-known provenance.

## Background

Archaeological ceramics can be studied in the context of origin of production or production technologies, as well as the distribution of specific ware types or whole assemblages [1-9]. Such studies have at their disposal an arsenal of different techniques, both analytical [10-16] and statistical [17-20], to arrive at answers to archaeological issues. Special place in a long list of analytical techniques belongs to non-destructive analyses performed using IR or Raman spectroscopy, PIXE or XRD, [21-26]. One of the non-destructive techniques that have been most commonly used is energy dispersive X-ray fluorescence (EDXRF) spectrometry proven to be efficient and suitable for archaeological ceramics provenience studies [4,5,15]. During the past ten years the use of portable XRF (PXRF, pXRF), field-portable (FPXRF) or handheld XRF spectrometers has increased significantly [27]. Such instruments (and consequently technique) become affordable for many applications that generate fast results which imply almost immediate interpretation and decision.

Different supervised as well as unsupervised multivariate statistical methods are widely and successfully used in archaeometric data analysis. Commonly applied methods include principal component analysis (PCA), various forms of cluster analysis (CA), and discriminant analysis (DA, both linear and quadratic), followed by more recent (neural network and fuzzy) methods [17], although the application of combined techniques has been reported in the literature [28]. Multivariate statistical methods can be used in provenience studies of artefacts [2,6], as well as for the recognition of local ceramic production and its characterisation, distinguishing from objects of possible trading activities [3], production dating [28], etc. Even so, discussion on applied dimension reduction technique regarding its validity from the aspect of information loss can be rarely found in the literature.

Systematic analytical examinations of archaeological ceramics from the Vinča culture are very obscure. As the ceramics belonging to the Vinča culture play an important role in global archaeology, it is of great importance to study as many aspects of their provenience as possible.

**Figure 1:**
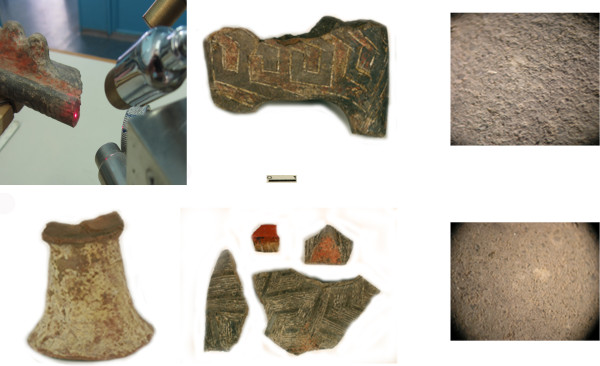
The pEDXRF equipment during the measurement, some representative ceramic fragments from the three archaeological sites and ceramic body OM picture.

The objective of this research was to examine the possibility of using information derived by pEDXRF spectrometry to classify ceramics. So, the question arises whether pattern recognition methods can be applied to the data obtained by this method in a way as in provenance studies. The focus of this study was on non-destructive characterisation of ceramic findings excavated at three Neolithic sites: Vinča-Belo Brdo near Belgrade, Pločnik near Prokuplje, and Bubanj near Niš, all in Serbia, and their classification according to elemental compositions and well-known provenance. There are a few points that must be emphasised. The ceramics were characterised by means of its elemental composition obtained using pEDXRF spectrometry. Thirty-two pottery sherds from the site of Vinča, 21 figurines or fragments of figurines, 4 fragments of altars, 2 pottery sherds from the site of Pločnik, and 15 pottery sherds from the site of Bubanj were organised in three sample assemblages. The dimensions of the ceramic sherds from the site of Vinča range from 10 × 5 cm (large pieces) to 5 × 3 cm (the small ones), and the average thickness is approximately 4–6 mm. The sherds have mostly black to grey ceramic body. In the archaeological layer of interest, several figurines had also been found, but only two of them were available for the analysis. The figurines from the site of Pločnik are generally about 10–15 cm high and 5 cm in diameter. Some of the figurines are larger, while others are much smaller, looking like amulets. The figurines, pottery sherds and altars have black ceramic body. The pottery sherds from the site of Bubanj have black and brownish to red ceramic body. The average dimension of the fragments from the site of Bubanj is 5 × 5 cm and their thickness is 5–10 mm (see Figure 1. A more detailed sample description can be found in [29]). The assemblages were composed of ceramics of different production quality (but with a homogenous structure, previously analysed by optical microscopy, which improved the absence of the tempers of considerable grain size and pores whose presences could affect homogenous elemental distribution around the examined surface) and usage (pottery, figurines, and altars). The main characteristic of the ceramic assemblages was their well-known provenance (on the basis of archaeological reasons [30,31]). Such an approach was selected since in archaeometry research, two different approaches can be followed to determine the origin of production: comparison with the clay or with the artefacts of well-known provenance as referred in [32].

Analytical examinations were followed by application of pattern recognition methods to the obtained results as part of the decision-making procedure developed and improved to classification (and consequently sourcing) purpose which has been described below.

## Results and discussion

### Non-destructive characterisation

The elemental compositions resulting from pEDXRF measurements of 67 investigated samples were used to form a training data set (TRS) as 67 × 10 matrix. The TRS comprised the intensity results reported as the average net peak area values for X-rays detected over 100 s of live counts for ten elements: Si, K, Ca, Ti, Mn, Fe, Zn, Rb, Sr and Zr, chosen so that net peak area uncertainty remained below 10% (as suggested in [33]). The uncertainty of the net peak area was usually much less than 10% for most of the selected elements, except for Mn and Zn where the uncertainty was 15% in some measurements. For Cr, Cu, Pb or Y, uncertainty did exceed the desirable 10% level in most of the measurements, or a large number of measured values were affected by poor counting statistics implying that those elements needed to be excluded from the TRS. According to published data [4,33,34], the selected elements can be considered as representative for classification purposes.

The test data set (TDS) was formed in the same manner. The same ten elements were measured under the same conditions as for the TRS, for eight additional ceramic sherds (2 from the site of Bubanj, 2 from the site of Pločnik and 4 from the site of Vinča) forming 8 × 10 matrix.

### Multivariate analysis and classification

Table 1 reports the elemental content of the ceramic sherds from three Neolithic sites. The net peak area mean value and standard deviation (SD) are shown for each element and each group (sampling location) and for the whole assemblage.

**Table 1:** Elemental composition of the three ceramic samples groups and the whole assemblage

**Variable**	**BUBANJ (n = 13)**	**PLOCNIK (n = 25)**	**VINCA (n = 29)**	**ALL (n = 67)**
	**Mean ± SD**	**Mean ± SD**	**Mean ± SD**	**Mean ± SD**
Si	14.46 ± 4.32	8.87 ± 3.85	13.38 ± 4.16	11.91 ± 4.68
K	55.01 ± 9.72	24.31 ± 9.48	37.89 ± 8.98	36.14 ± 14.44
Ca	40.61 ± 12.30	44.00 ± 28.05	56.38 ± 21.44	48.70 ± 23.57
Ti	44.86 ± 11.10	29.19 ± 11.69	37.26 ± 8.82	35.72 ± 11.78
Mn	13.61 ± 6.57	13.47 ± 12.37	10.45 ± 7.23	12.19 ± 9.38
Fe	1223.56 ± 249.18	789.64 ± 280.00	976.99 ± 197.44	954.92 ± 284.80
Zn	14.30 ± 15.79	8.31 ± 5.81	28.21 ± 42.96	18.09 ± 30.41
Rb	12.41 ± 3.46	7.86 ± 2.88	10.18 ± 2.95	9.75 ± 3.42
Sr	14.98 ± 5.72	11.03 ± 3.15	14.17 ± 4.19	13.16 ± 4.45
Zr	19.47 ± 6.16	15.15 ± 6.28	23.62 ± 5.43	19.65 ± 6.95

The mean intensity is expressed in counts per second (cps) and n denotes the number of samples in each group. An additional table shows dataset in more detail [see Additional file 1].

**Figure 2:**
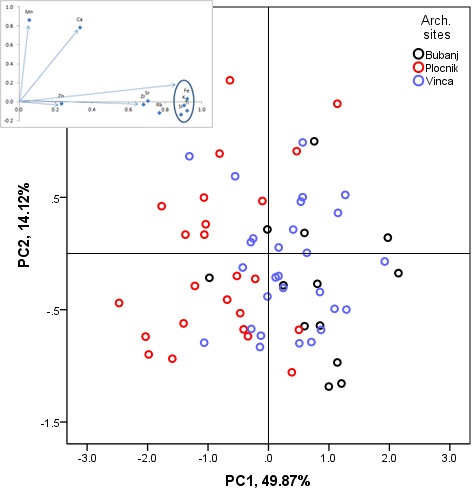
A score and loadings plot of the first two PCs of the pEDXRF data for Neolithic ceramics.

The results of PCA based dimension reduction (performed in MATLAB - version R2010a, Math Works, Inc. environment and using IBM SPSS Statistics 19, software package, both also used for all other calculations) are presented in Table 2 and Figure 2. Table 2 shows the principal component (PC) scores for the first two PCs and the variance explained by each of them. The first three PCs accounted for more than 75% of the variance in the TRS, where PC1 explains 49.87% and PC2 explains 14.12% of the variance. The PC loadings indicate that Fe, Ti and K, dominate the first PC, respectively, while Mn and Ca are the most dominant parameters in the second PC. The scatter-plot of the first two PCs (Figure 2) represents three statistical groups. None of them is clearly separated along the PC axes to satisfy the required classification. Elements such as Fe, Ti and K have high loading values (Table 2) and can be underlined as elements of important variability. In this context, it may be concluded that some of the information has been lost, in dimension reduction procedure that concerns origin of production. High loading value for Mn might arise from poorer counting statistics but also provide a clay geochemical signature since it tends to concentrate in clay fraction. This result may indicate the clay sources or possible technology used for ceramic manufacturing. High loading value for Mn, strongly correlated with a high variance of Ca along PC2 axis indicate the influence these elements on within-group cohesion (see Figure 2). Group spreading could be caused by that variance in Mn and Ca. This implies a good knowledge of the clay properties (it can be assumed that the used clay contained homogeneously distributed fine-grained CaCO_3_ particles, which provided easier sintering) and the particular clay sources that were chosen.

**Table 2:** First two PCs of the training dataset: eigenvalues, explained and cumulative variance, and loadings of the variables

**PC**	**Initial eigenvalues**			**Factor loadings**									
	**Total**	**% of Variance**	**Cumulative %**	**Si**	**K**	**Ca**	**Ti**	**Mn**	**Fe**	**Zn**	**Rb**	**Sr**	**Zr**
1	4.987	49.875	49.875	.884	.901	.337	.917	.055	.918	.232	.766	.703	.678
2	1.412	14.120	63.995	-.139	-.045	.789	-.106	.859	.019	.023	-.129	.022	.013

**Figure 3:**
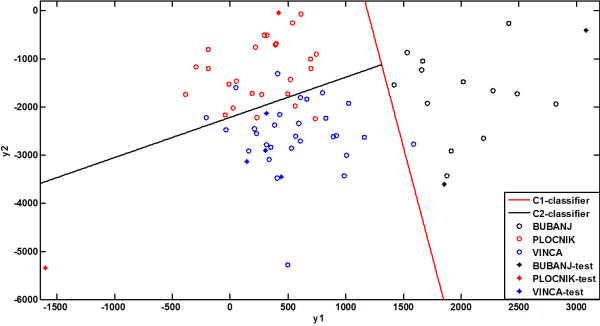
Classification results: linear classifiers and test samples shown together with classified training samples.

Implementing scattering matrices-based dimension reduction, the feature vectors from the TRS were projected from a 10-dimensional into a two-dimensional space, taking care to minimise information loss (Figure 3). The newly formed two-dimensional space is defined by a linear combination of the original features, i.e. two new features (Feature 1 denoted by *y1* and Feature 2 denoted by *y2*) were extracted. As dimension reduction was performed in an optimal way, the extracted features *y1* and *y2* can be considered as the best features for classification purposes [35]. The dependence of *y1* and *y2* on the original features and the influence of the original features on class separability (i.e. classification) are shown in Table 3, indicating that K are Zr are the most responsible for class separability along the y1-axis, while Zr and Si have the most important influence on class separability along the y2-axis, respectively. Group cohesion is best preserved for the Pločnik and Vinča groups, while for the Bubanj group this cohesion is more disturbed. As the ceramic samples from the site of Vinča and Pločnik date from two very close periods (first half of the fifth millennium BC) this result may indicate similar technology used for ceramic manufacturing. The ceramic samples from the site of Bubanj, was tentatively dated to the end of the seventh millennium BC (Starčevo group) and the second half of the fifth millennium BC (Bubanj-Hum I), what might be the reason of decreases group cohesion caused by some difference in production technology.

**Table 3:** **Dependence of extracted features *y1 *and *y2 *on original features**

**Original feature**	**y1**	**y2**
Si	−0.31	**−0.42**
K	**0.69**	−0.18
Ca	−0.10	−0.20
Ti	−0.35	0.07
Mn	0.04	0.37
Fe	0.01	0.01
Zn	0.01	−0.07
Rb	0.36	−0.002
Sr	0.08	0.04
Zr	**−0.39**	**−0.78**

Following dimension reduction, it was possible to classify the reduced vector (newly formed vector $Y={\left[y_{1}\;y_{2}\right]}^{T}$), into one of the three classes (Bubanj, Pločnik or Vinča excavation site). This paper presents a hierarchical classification method based on one sequentially chosen class out of two classes. The three classes presented in Figure 3 are not quite separable from each other (especially the classes representing the sites of Pločnik and Vinča). The third class (representing the site of Bubanj) is separated from the other two in such a way that it is possible to determine the linear segments which differentiate from the two patterns without any classification error. In this case proper classification has been achieved by designing a linear classifier, based on the desired output (*h*_*1*_*(Y)*). Following the demand that the decision – making procedure should be rapid, simple and effective in classification of unknown samples, it is reasonable and justifiable to perform the second classification of the Vinča and Pločnik classes using the simplest classifier of the linear discriminatory function type. The two linear classifiers based on the desired output are designed and their dependence on measured variables is as follows (in matrix representation as more convenient):

$$h_{1}\left(Y\right)=V_{1}^{T}Y+v_{01}=\left[\begin{array}{cc} 0.0009 & 0.0001 \end{array}\right]Y-1.0912$$

$$h_{2}\left(Y\right)=V_{2}^{T}Y+v_{02}=\left[\begin{array}{cc} -0.0006 & 0.0007 \end{array}\right]Y+1.5116$$

It is now necessary to decide whether the new vector *Z* belongs to the Bubanj excavation site or not. If it does not belong to Bubanj (*h*_*1*_ will have a negative value), the next step is to choose between the Pločnik and Vinča excavation sites (*h*_*2*_ positive value indicates the Pločnik site while the negative value of *h*_*2*_ indicates that the analysed sample belongs to the Vinča group). The classification results are presented in Table 4. It is apparent that ceramic sherds from the site of Bubanj are 100%, from the site of Pločnik 88%, and from the site of Vinča 86.2% properly classified. The recognition ability of the present classification is 89.6% of correctly classified samples of the TRS. Note that a design of more complex classifiers (quadratic, for example), instead of the linear classifier proposed in this paper, would certainly improve the efficiency of the classification. However, the chosen linear classifier seemed to be the most convenient type of classifiers because it not only provides an objective and simple procedure, which addresses all available measurement data in a specific way and makes a decision based on these data, but also allows a deep insight into the ceramic assemblages. The relative position of the points representing ceramic samples in two-dimensional space from the classification line can be of importance in detecting possible trading activities, production technology or even measurement irregularities (due to in-situ conditions).

**Table 4:** Classification results for the three site groups and leave-one-out cross validation results

			**Predicted Group Membership**			**Total**
			**Bubanj**	**Pločnik**	**Vinča**	
TRS	Count	1	13	0	0	13
		2	0	22	3	25
		3	1	3	25	29
	%	1	**100**	0	0	100
		2	0	**88**	12	100
		3	3.4	10.3	**86.2**	100
Cross validated	Count	1	10	1	2	13
		2	0	18	7	25
		3	1	5	23	29
	%	1	**76.9**	7.7	15.4	100
		2	0	**72**	28	100
		3	3.4	17.2	**79.3**	100

The success of the classification model was tested by the leave-one-out cross validation method [36]. Only analysed cases were cross validated, and each case was classified using the functions derived from all cases other than that case. The achieved prediction ability was 76.1% of cross-validated grouped cases correctly classified. Another test of the classification model was performed. Two (*h*_*1*_ and *h*_*2*_) linear classifiers designed in the training step were used for the classification of the eight vectors belonging to the TDS. The results (Figure 3) show that only one sherd from the TDS was not correctly classified using the model developed during the training process.

## Conclusions

According to the results presented, several conclusions can be drawn. Algorithm of the proposed decision-making procedure enables effective classification of ceramic artefacts based on their elemental compositions determined by pEDXRF spectrometry. As shown, the data from the first algorithm step, denoted as in-situ data acquisition, can be used as a viable tool for sourcing ceramics although their accuracy may not be the same as in the case of other methods used for the purpose (e.g. ICP, NAA, PIXE, or laboratory XRF).

The step in algorithm, denoted as dimension reduction gave significant results rarely discussed in the literature. The results derived by PCA dimension reduction show that the elements which contribute the most to the formation of the PCs are not quite informative for classification as well (also confirmed by biplot examination). In other words, reliable classification of ceramics in a space determined by the greatest variance in their elemental compositions is not feasible with the data obtained by pEDXRF characterisation. This outcome underline that the selection of the greatest variance in addressing a new space can lead to a loss of information carried by the data.

On the other hand, it is possible to achieve the initial goal (expressed through the classification of ceramics based on the elemental composition) by a method founded upon dimension reduction, which has scattering matrices as its basis and which takes into account minimal information loss. According to the results obtained it is safe to say that the success of classification, expressed through prediction and recognition ability, allows the application of this method for the identification of objects based on their well-known provenance and that the proposed decision-making procedure yields satisfactory classification results. It should be emphasized that the selection of dimension reduction technique also has to be careful and in accordance with the aim of data analysis.

There are no previous studies dealing with the investigation of elemental patterns of ancient pottery from the Neolithic sites in Serbia therefore no comparison can be made. The results of the present study can support provenience study issues, in developing a compositional databank and establishing reference groups of pottery from Neolithic sites. An ongoing analysis of sherds from the other sites is expected to provide additional insight into pottery making techniques, trade and cultural exchange in the region.

The conclusion that should be emphasized, based on the results obtained, is that pEDXRF spectrometry when used in investigation of the origin of ceramic artefacts can provide viable results by carefully selecting the experimental conditions and well-thought-out procedure of data processing. This conclusion is particularly important in cases when it is not possible to apply the methods with high precision and sensitivity for determination of elemental compositions, although they have been proven to be very successful in meeting the requirements related to the determination of the artefact origin, either because of their destructiveness or non-portability.

## Methods

### Experimental

pEDXRF analysis for non-destructive and non-invasive characterisation of the ceramic artefacts was performed using a milli-beam spot XRF spectrometer. The spectrometer (in-house developed at the Vinča Institute of Nuclear Sciences, Belgrade) is based on an air cooled X-ray tube (Oxford Instruments, Rh-anode, max 50 kV, 1 mA) with a pin-hole collimator and a SiPIN X-ray detector (6 mm^2^/500 μm, Be window 12.5 μm thickness), associated with a DSP (X123, Amptek, Inc.) for spectra acquisition. Two laser pointers were used for proper positioning of the analysed sample in the cross-point of the exciting X-ray beam and the detector axis, respectively. ADMCA software was used for spectra analysis. A 35 kV high voltage, 800 μA, no filter and a 100 s measuring time were selected as experimental parameters and kept constant during all measurements. The geometry parameters were chosen as follows: detector-sample distance = 21 mm, X-ray tube-sample distance = 16 mm, detector-X-ray tube angle = 45° and sample-X-ray tube angle = 90°. Instead of quantification, it was presumed (similarly to [33]) that the high correlation coefficient (*R*^*2*^) values obtained (ranging from 0.863 for K to 0.994 for Fe) between average net peak area values and selected element concentrations for powdered CRM (NIST SRM-2711 Montana soil, NCS CRM DC 73301 rock) and RM (IAEA XRF-PT China ceramic and lake sediment) can also be achieved in ceramic fragments analysis.

The measuring areas of all the samples were polished and cleaned before analysis. Each sample was analysed in three points, as it was suggested in [33], and the average net peak area values were considered. Whenever possible, different sample fractured sides were selected for measurement. In other cases, the measurements were performed at the most distant spots, providing in this way the representativeness of measurements.

### Pattern recognition methods and decision-making procedure

As already stated, the use of in-situ EDXRF spectrometry for non-destructive characterisation of ceramic artefacts generates data whose quick interpretation is an increasingly frequent requirement [37]. To meet this requirement, it is useful to design an efficient and reliable decision-making procedure [38]. This paper presents one such procedure consisting of the following steps: a) in-situ data acquisition; b) generation of vector *X*; c) dimension reduction; d) classifier design and e) classification followed by classification success testing.

During the analytical examination and characterisation of ceramic sherds, the elemental composition was determined as described above. The result was that 67 different ten-dimensional vectors were generated. This provided a considerable amount of data which did not need to be equally informative for the characterisation of ceramics or the determination of their provenance, and it was therefore necessary to separate those parameters which carry the most information about the characteristics or provenance. The first step towards this goal was to make the performed measurements “more visible”. The pattern recognition theory has developed techniques to address this issue referred to as dimension reduction. The main goal of dimension reduction is to project the original vector *X* of dimension *n* onto a vector *Y* of dimension *m* (considerably smaller than the initial dimension *n*) in such a way as to minimise the loss of information. Two approaches were chosen to reduce the initial 10-dimensional space to 2-dimensional space: PCA and scattering matrices-based dimension reduction, described below in more detail. Dimension reduction is a step in the decision-making procedure, followed by classifier design and then classification. The design of a proper classifier is a procedure dependent on the previous step, but it is desirable to choose a procedure as simple and as fast as possible, which will achieve the best classification results at same time.

PCA, also known as Karhunen–Loeve transform, is a widely used method for dimension reduction. The purpose of PCA is to project n-dimensional data onto a lower d-dimensional subspace in a way that maximises the variance [39-42]. The derived new uncorrelated variables that are linear combinations of the original one result in finding of a smaller group of underlying variables that describe the data. The first few components will account for most of the variation in the original data, but they may not be able to accurately represent group membership [35,40].

As the dimension reduction of the original space is only one step in the procedure whose goal is classification, the scattering matrices-based dimension reduction method was tested as the most appropriate choice. The main advantage of dimension reduction performed in such way as to preserve class membership is two-fold. First, in low-dimensional space it is possible to visualise the classification results and choose the appropriate classifier design approach. Second, it is possible to identify the important measurements with regard to classification. Dimension reduction consists of finding a transformation matrix *A* (*Y = A*^*T*^*X*) which will reduce the original data space (*X*) dimensionality in the new (*Y*) one, considerably lower dimensionality. The optimal transformation matrix *A* is the explicate solution of the optimisation criterion $J=tr\left(S_{w}^{-1}S_{b}\right)$, obtained as the solution for the generalised singular value decomposition of the matrix $S_{w}^{-1}S_{b}$ (where *S*_*w*_ and *S*_*b*_ represent the within-class scatter matrix and between-class scatter matrix, respectively). The *m* eigenvectors correspond to the *m* largest eigenvalues form the matrix *A*[35]. Two-dimensional projection is the most desirable, allowing examination of the classification results in terms of recognition ability (percentage of members of the training set correctly classified) and prediction ability (percentage of members of the test set correctly classified using the rules developed during the training).

## Competing interests

The authors declare that they have no competing interests.

## Authors' contributions

MGK conceived of the study and, together with MMS, participated in its design and drafted the manuscript. MMS coordinated ceramic sherds sampling. MGK, MMS and VA participated in all analytical procedures. MGK and GK took part in the design and performed the statistical analysis. This project was based on the ideas and carried out under the guidance of MGK, MMS and GK, in consultation with RJH. All authors have read and approved the final manuscript.

## Supplementary Material

Additional file 1 **Elemental composition of the three ceramic samples group. **The mean intensity is expressed in counts per second (cps). Corresponding measurement uncertainties are reported in the brackets. Click here for file
